# 

**Published:** 1915-10-23

**Authors:** 


### CONTENTS.

PAGE

**The Treatment of Wounds Controversy** ... 61
**The Universities and Medicine** ... ... ... 62
**Hospital and Institutional News** ... ... ... 63
The Execution of Miss Cavell—The Norwich Vacancy—The Recruit's Heart—What is a War Hospital?—Manchester's New Home for Epileptics—Trouble at the Endsleigh Palace Hospital—War Hospital Expansion at Manchester—Free Choice of Doctor—The Mental Hospital's Roll of Honour—Working-Class Subscriptions in War-Time—The Cook's Opportunity—Drug Economy in Poor-Law Infirmaries—The Closing of Poor-Law Institutions—Hospitals and the Choice of Disinfectants—Medical Education in Wales—A Matrimonial Scheme—The Position of the R.N.V.R. Surgeons—The Grievance Admitted—The Economics of Unpaid Employment—Ballahouston Hospital Opened—Cardiac Disease in Soldiers—Southwark Military Hospital—The Medical Curriculum in Australia—Exploiting the Memory of the Dead—Panel Practitioners' Accounts—This Week's Drug Market.
**The War and the Wounded** ... ... ... ... 69
Hospital Provision: Its Difficulties and Limitations.

PAGE

**The Territorial Hospital** ... ... ... ... 71

I.—Administrative and Medical Staff.

II.—The Nursing System: Some Difficulties and Differences.

**Poor-Law Buildings and Military Hospitals** ... 75

**Temporary Hospitals Construction** ... ... 77

Some Plans, Practical and Otherwise.

**The Temporary War Hospital** ... ... ... 78

Attached to the General Hospital, Nottingham.

**The Third London General Hospital** ... ... 79

Wandsworth Common, S.W.

**The Fifth Northern General Hospital, Leicester** 81

The Recent Additions.

**War Influences on Medical Staffs and the Profession** ... ... ... ... ... ... 83

**Obituary** ... ... ... ... ... ... ... ... 84

**Poor-Law Infirmaries in War-Time** ... ... 85

How Difficulties have been Surmounted.

**The Red Cross and St. John's Organisation** ... 86

The Joint Societies' Varied Field.

**Patriotism and the Nurse-Training Schools** ... 87

I.—Examples in Great Britain and Ireland.

**Some General Effects of War** ... ... ... 89

Upon the Voluntary Hospitals.

[*See over.*]

### CONTENTS—(*continued*).

| | PAGE |
|---|---|
| **Parliament and the Profession** ... ... ... | 90 |
| **Passing Events and Topics** ... ... ... | xviii |
| **The Editor's Letter-Box** ... ... ... | xviii |
| **The Institutional Worker** ... ... (Suppl.) | 1 |
| The Effect of the War upon the Supply of Surgical Requisites. | |
| Question for October. | |
| Questions Invited. | |
| Enquiries and Answers. | |
| War Employment. | |
| Employment and Training. | |
| The Housekeeper's Department. | |

